# Radiolabelled polymeric IgA: from biodistribution to a new molecular imaging tool in colorectal cancer lung metastases

**DOI:** 10.18632/oncotarget.19616

**Published:** 2017-07-27

**Authors:** Helene Carpenet, Armelle Cuvillier, Aurélie Perraud, Ophélie Martin, Gaël Champier, Marie-Odile Jauberteau, Jacques Monteil, Isabelle Quelven

**Affiliations:** ^1^ Nuclear Medicine Department, Dupuytren University Hospital, 87042 Limoges, France; ^2^ EA 3842 – Cellular Homeostasis and Diseases, Faculty of Medicine, University of Limoges, 87025 Limoges, France; ^3^ B Cell Design SAS, 87000 Limoges, France; ^4^ UMR CNRS 7276 – CRIBL, University of Limoges, 87025 Limoges, France

**Keywords:** anti-CEA IgA, radiolabelling, mucosal biodistribution, metastases, colorectal cancer imaging

## Abstract

By radiolabelling monomeric (m) and polymeric (p) IgA with technetium 99m (^99m^Tc), this study assessed IgA biodistribution and tumour-targeting potency. IgA directed against carcinoembryonic antigen (CEA), a colorectal cancer marker, was selected to involve IgA mucosal tropism.

Ig was radiolabelled with ^99m^Tc-tricarbonyl after derivatisation by 2-iminothiolane. ^99m^Tc-IgA was evaluated by *in vitro* analysis. The biodistributions of radiolabelled anti-CEA mIgA, pIgA and IgG were compared in normal mice. Anti-CEA pIgA tumour uptake was studied in mice bearing the WiDr caecal orthotopic graft.

IgA radiolabelling was obtained with a high yield, was stable in PBS and murine plasma, and did not alter IgA binding functionality (Kd ≈ 25 nM). Biodistribution studies in normal mice confirmed that radiolabelled pIgA – and to a lesser extent, mIgA – showed strong and fast mucosal tropism and a shorter serum half-life than IgG. In caecal tumour model mice, evaluation of the anti-CEA-pIgA biodistribution showed a high uptake in lung metastases, confirmed by histological analysis. However, no radioactivity uptake increase in the tumoural caecum was discerned from normal intestinal tissue, probably due to high IgA caecal natural tropism. In microSPECT/CT imaging, ^99m^Tc-IgA confirmed its diagnostic potency of tumour in mucosal tissue, even if detection threshold by *in vivo* imaging was higher than *post mortem* studies. Contribution of the FcαRI receptor, studied with transgenic mouse model (Tsg SCID-CD89), did not appear to be determinant in ^99m^Tc-IgA uptake.

Pre-clinical experiments highlighted significant differences between ^99m^Tc-IgA and ^99m^Tc-IgG biodistributions. Furthermore, tumoural model studies suggested potential targeting potency of pIgA in mucosal tissues.

## INTRODUCTION

Colorectal cancer (CRC) is the most commonly diagnosed cancer worldwide. For the past few years, almost 1.4 million new cases have been diagnosed every year (representing 9.7% of all cancers), with 450,000 new cases being reported in Europe (where CRC is the second-most common cancer, representing 13% of all cancers). CRC is also the second-most common cause of cancer-related deaths in Europe, with an estimated 215,000 deaths every year. Cancer survival rates are based on initial staging of the cancer at diagnosis. Optical colonoscopy remains the gold-standard investigation for early detection of CRC. Accurate staging remains critical for determining the most relevant treatment, and also offers prognostic and outcome indicators. However, despite the above, according to a review by Bradley no definitive consensus on the optimal imaging staging strategy has yet been established for CRC [1]. More specific and sensitive non-invasive methods are needed. One such method is immunoscintigraphy, a useful technique that has continued to expand since the 1980s commensurate with monoclonal expansion. Monoclonal antibody (mAb) generation in 1975 by Köhler and Milstein combined to nuclear imaging technology progress led to development of monoclonal antibodies labelled with radioactive isotopes to target tumours [2]. Concerning staging of CRC, the main interest in immunoscintigraphy lies in its sensitivity for detecting distant metastasis.

Many antibodies or antibody fragments have been developed for detecting primary tumours and distant metastasis. In cases of cancer with mucosal localisation, satumomab (Oncoscint^®^) directed against glycoprotein TAG-72 radiolabelled with 111-Indium has been used in the US since 1992 for CRC diagnosis [3]. Arcitumomab (CEA-Scan^®^), a F(ab')_2_ directed against carcinoembryonic antigen (CEA) labelled with 99m-Technetium (^99m^Tc), received FDA and EMEA approval in 1996 for diagnosis of CRC recurrence [4, 5]. Another ^99m^Tc-radiolabelled Fab, nofetumomab (Verluma^®^) – directed against pancarcinoma glycoprotein antigen epithelial cell adhesion molecule (EpCAM) – was FDA-approved for the diagnosis of small cell lung cancer. Today, satumomab is the only mAb still used in the US for the immunoscintigraphy of mucosal tumour localisation. Their diagnostic value remains controversial due to the low contrast between normal and tumoural tissue, especially compared with 18-fluoro-deoxyglucose positron emission tomography (FDG-PET) [6]. However, FDG is not a specific tracer as high FDG uptake in inflammatory or infectious lesions, and variable physiological concentrations in normal tissues/organs, can be confused with malignant tissue [7]. An approach to overcome the limitations of FDG-PET involves targeting molecules to localise specific tumour markers, with preferential tropism for the tissues of interest, such as mucous membranes.

Antibodies directed against CEA appear to be good candidates to target CRC. Indeed, CEA, a 180-kDa glycoprotein, is part of a large family of 12 related members, whose functions can vary widely and include cell adhesion, participation in intracellular signal cascades, cell migration, inflammation, and angiogenesis. CEA is synthesised mainly by the digestive tract and is polarised at the apical pole in the epithelial wall. This oncofetal antigen is quite absent from healthy adult tissues. CEA is frequently expressed, with a high serum concentration, in adenocarcinomas of various origins, such as breast, medullary thyroid, lung, and ovarian cancers, as well as CRC. CEA was identified in CRC for the first time in 1965 [8]. Its expression is upregulated in approximately 90% of advanced CRC cases [9]. One in two patients diagnosed with CRC already has metastasis, thereby negating the use of a first-line surgical strategy; furthermore, many cases do not respond to chemotherapy. In such advanced cases, the prognosis is very poor, and the average survival time is 5 to 9 months. Although not a specific marker of CRC, CEA has prognostic value in patients with metastatic cancer. It is elevated in 58% to 65% of cases, and the first sign of recurrence can precede clinical or radiological recidivism by 1.5 to 6 months. Several clinical studies are underway to assess the relevance of targeting CEA by immunoscintigraphy for the detection of primary tumour and metastasis [10, 11]. Nevertheless, published data are only based on the Immunoglobulin (Ig) G or IgG fragment, and no study has used IgA. In humans, IgA is the most heavily produced isotype (66 mg/kg/day) and the second-most prevalent circulating isotype, after IgG. Long regarded as an anti-inflammatory antibody involved in maintaining homeostatic balance at the level of the mucous membrane, it has been demonstrated recently that IgA can enable or inhibit different inflammatory responses [12]. IgA efficacy to recruit effector cells, such as monocytes, macrophages, granulocytes and Kupffer cells, was demonstrated for antibody dependent cell-mediated cytotoxicity (ADCC) [13]. IgA interaction with myeloid cells is mediated with the IgA Fc receptor, FCαRI (CD89). Importantly, mice lacking to express any human FCαRI homologue, a transgenic mouse model (Tsg SCID-CD89) was described to express human FCαRI on mouse myeloid lineage [14] and mimics effector cells recruitment and cell cytotoxicity mechanisms observed in human [15, 16] in presence of human or humanised IgA.

IgA is expressed in three different forms: monomeric (mIgA) (in the blood: 1 to 3 g/L), dimeric (dIgA)/polymeric (pIgA) (in mucous membranes) and secretory (SIgA) (in the mucosal organs). The B lymphocytes are present in the serous compartment of mucosal organs and predominantly produce dIgA (two molecules of IgA complexed by the J chain peptide). The dimers, specifically recognised by the polymeric Ig receptor (pIgR) expressed on the basal side of epithelial cells, are endocytosed and translocated to the apical side of the cells. At the time of exocytosis, the extracellular part of the pIgR is cleaved and remains complexed to the dimer, thereby forming the secretory component, the SIgA [12]. Moreover, dIgA has a cell signalling capacity greater than that of monomeric IgA because the dimer carries twice as many paratopes [16]. Knowledge of IgA and its applications is limited partly due to difficulties in the identification of IgA-producing B cells, and with respect to stable production of IgA antibodies. IgA-secreting B lymphocytes represent less than 1% of normal mouse splenocytes (even fewer are found in mucosal lymphoid compartments: 0.01% in the *lamina propria* and 0.1% in Peyer’s patches) [17]. The recently developed HAMIGA™ technology allows this limitation to be bypassed [18]. By replacing the Sμ domain with a human alpha 1 constant gene downstream of variable gene segments, the population of IgA-secreting lymphocytes B in the spleen was increased significantly (62% of B220^+^ membrane IgA cells in the spleen of HAMIGA™ mice versus undetectable level in wild-type mice) [18] which allows to easily sort highly specific monoclonal humanised IgA. From immunised transgenic human α1 mice, IgA1 were produced in hybridoma, B lymphocyte hybrid cells, using its dedicated glycosylation pathways of antibodies.

Concerning IgA application in nuclear medicine, ^99m^Tc remains the most widely used isotope for diagnosis, due to its suitable nuclear and chemical properties, and good availability [19]. This radionuclide has favourable physical characteristics for high-efficiency detection in molecular imaging (with a γ-ray of 140 keV) and for radiation protection due to a short half-life (T_1/2_ = 6h). This short half-life is not optimal for IgG (serum half-life 20 days) radiolabelling but seems suitable considering the shorter half-life of IgA (3–6 days). In a previous study, we showed that indirect radiolabelling using a bifunctional chelating agent, such as the tricarbonyl core [Tc(CO)_3_(H_2_O)_3_]^+^, and a spacer such as 2-iminothiolane (2-IT), provides good radiolabelling yields with high specific activity, and preserves IgG structure and immunoaffinity with limited amounts of antibody [20].

Here, by radiolabelling with ^99m^Tc, we report the natural pharmacokinetic and biodistribution of different isotypes of antibodies, IgA directed against CEA, compared with IgG in healthy mice and in a xenograft mouse model of human colon carcinoma. This model enables us to evaluate the mucosal tropism of IgA antibodies and to test the ability of IgA antibodies, directed against a well-characterised human tumour-associated antigen, to detect early-stage mucosal metastasis foci. Biodistribution was firstly evaluated by *post mortem* organs counting. This most sensitive technique is the gold standard to evaluate accurately molecule biodistribution, but needs animals sacrifice. MicroSPECT/CT studies, which are less sensitive but allow *in vivo* monitoring, were compared to gold standard method. Using the transgenic mouse model expressing the high affinity receptor FCαRI (Tsg SCID-CD89), we also evaluate the contribution of FCαRI in the distribution and the capacity of IgA to detect tumour *in vivo* by microSPECT/CT imaging.

## RESULTS

### IgA radiolabelling and ^99m^Tc-IgA-SH *in vitro* stability

**Figure 1:**
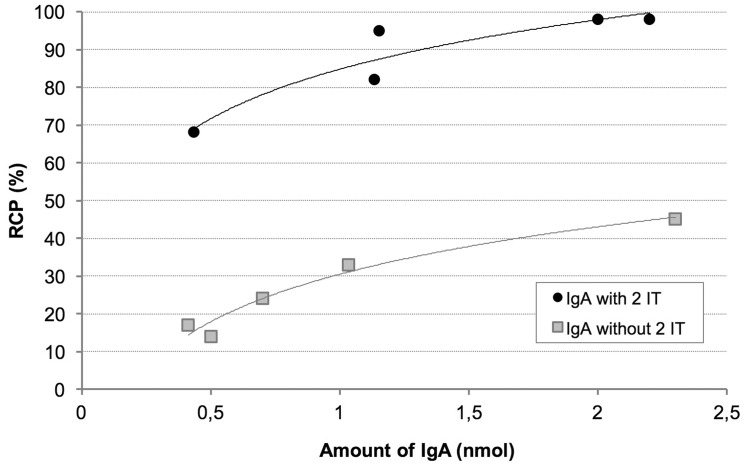
Comparison of native and 2-IT derivatised radiolabelled IgA yields according to IgA amount Radiochemical purity (RCP) analysis was performed using instant thin layer chromatography–silica gel (ITLC-SG)/normal saline.

To optimise IgA radiolabelling with the tricarbonyl core, increasing quantities of antibody were tested by derivatisation with 2-IT (Figure 1). Native and functionalised IgA (IgA-SH) radiolabelling yields were correlated with the IgA amounts. The maximum quantity usable prior to IgA precipitation (maximum concentration = 2 mg/mL) is 2.2 nmol of IgA. With this amount of native IgA, only 45% radiochemical purity (RCP) was reached. With derivatised IgA, much higher yields were achieved (RCP = 98%) for this maximum IgA quantity, and an RCP > 85% was obtained from only 1 nmol of IgA. These results confirmed that the derivatisation of IgA is necessary to obtain high radiolabelling of RCP. After determining the sulphydryl groups by using the micromethod and Ellman’s reagent, a correlation between the radiolabelling yields and number of thiol moieties on IgA was clearly seen, confirming derivatisation (data not shown). With derivatised IgA, on average, 1.7 ± 0.4 (*n* = 3) sulphydryl groups were grafted to each IgA molecule; with native IgA, the number was below the limit of quantification. With these optimised conditions (1.4 nmol of derivatised IgA), radiolabelling yields > 96.6 ± 2.1% (*n* = 13) were obtained. The specific activity corresponded to 115 MBq/nmol (511 MBq/mg).

Stability testing of ^99m^Tc-IgA-SH was performed over 24 h in phosphate-buffered saline (PBS), with storage at 4°C or 25°C, and in murine serum (1/10 dilution) at 37°C. In PBS, no appreciable loss in RCP was seen (at 24 h: ∆RCP = 4% and 8%, at 4°C and 25°C, respectively). However, after incubation in murine serum (1/10 dilution) at 37°C, a gradual decrease in radiolabelled IgA RCP was observed (at 18 h and 24 h: ∆RCP = 13% and 23%, respectively).

### Integrity of ^99m^Tc-IgA-SH

**Figure 2:**
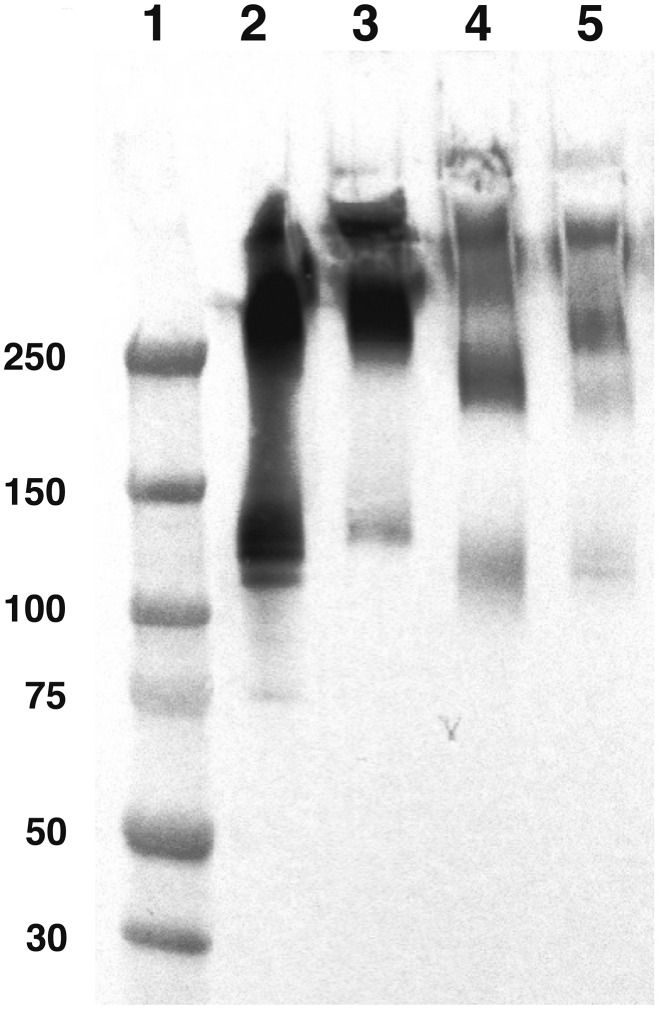
Molecular structure comparison of unlabelled and radiolabelled anti-carcinoembryonic antigen (CEA) or irrelevant monomeric and polymeric mixture (mpIgA) Anti-CEA or irrelevant mpIgA molecular structure was evaluated by Western blotting. Molecular weights in kDa are specified on the left. 1. Molecular weight Marker, 2. Unlabelled anti-CEA mpIgA-SH (monomeric ≈ 150 kDa and polymeric > 250 kDa) (1.5 mg), 3. Unlabelled irrelevant mpIgA-SH (1.5 mg), 4. Radiolabelled anti-CEA mpIgA (1.5 mg), 5. Radiolabelled irrelevant mpIgA-SH (1.5 mg).

To demonstrate full preservation of molecular integrity during the radiolabelling process, unlabelled and radiolabelled IgA samples of monomeric and polymeric mixture (mpIgA) were analysed by Sodium dodecyl sulphate-polyacrylamide gel electrophoresis (SDS-PAGE) (Figure 2).

Western blot profiles of mpIgA showed major bands at 120–140 kDa and 300–400 kDa, corresponding to the molecular weight of monomeric and polymeric forms, respectively, for both unlabelled IgA (lanes 2–3) and radiolabelled IgA (lanes 4–5). As expected, the molecular structure of IgA was preserved after radiolabelling; no low-molecular-weight band corresponding to free heavy chain release appeared for either radiolabelled antibody. A slight decrease in intensity was observed between unlabelled and radiolabelled antibodies due to Ig loss during the purification after incubation with 2-IT.

### Affinity of ^99m^Tc-IgA-SH

**Figure 3:**
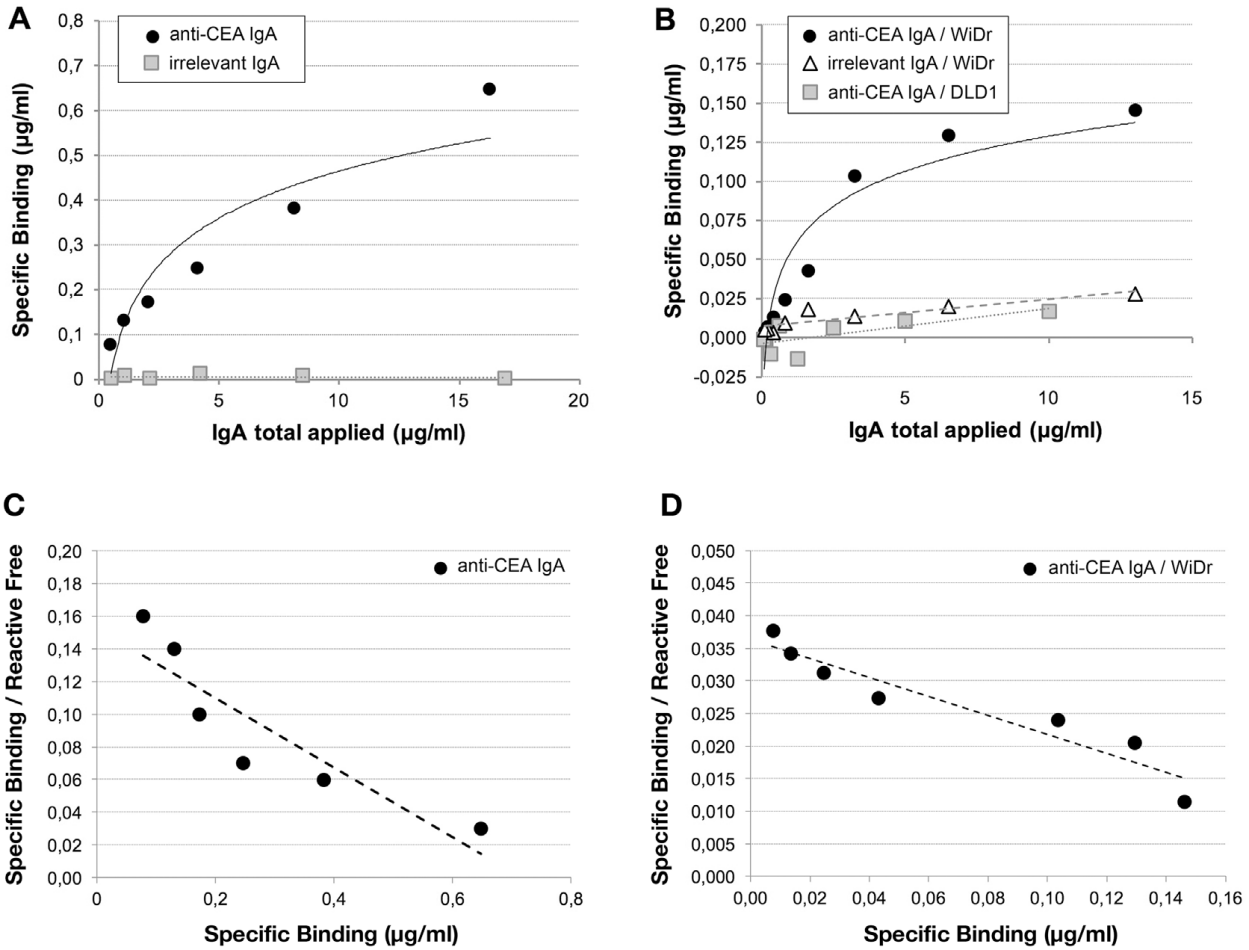
*In vitro* binding of ^99m^Tc-anti-CEA IgA-SH and ^99m^Tc-irrelevant IgA-SH Radiolabelled IgA specific binding was evaluated by RIA protocol **(A)** and on cells **(B)**. For cells studies, WiDr (CEA pos) and DLD1 (CEA neg) cell lines were tested. For the two experiments, Kd and Bmax were evaluated by a Scatchard plot (**C**: RIA; **D**: cells).

Immunoaffinity evaluation by radioimmunoassay (RIA) allowed us to observe that the radiolabelled anti-CEA IgA-SH has a high affinity for the target antigen (Figure 3A). The specific binding of ^99m^Tc-anti-CEA IgA-SH was confirmed by displacement with unlabelled anti-CEA IgA. The dissociation constant (Kd) and maximum number of antibody bound of ^99m^Tc-anti-CEA IgA-SH were estimated using a saturation binding assay and Scatchard analysis (Figure 3C). The assays provided a value of 20.3 nM for the Kd and 3.2 nM for the binding capacities (B_max_). Specific binding was also confirmed by lack of fixation of an irrelevant IgA (^99m^Tc-anti-peanut IgA-SH).

The immunoaffinity of ^99m^Tc-anti-CEA IgA-SH was also assessed using a saturation binding assay on the WiDr colon adenocarcinoma cell line, which expresses the fully folded membranal antigen (Figure 3B). This assay showed specific saturable binding. Using the Scatchard representation data, the calculated Kd and B_max_ were 30.5 nM and 1.2 nM, respectively (Figure 3D). The controls were performed with ^99m^Tc-anti-CEA IgA-SH on non-expressing CEA cells (DLD1; another colon adenocarcinoma cell line) and with irrelevant IgA on WiDr cells. As expected, no binding was observed under either of these conditions.

### Biodistribution of ^99m^Tc-anti-CEA IgA-SH and ^99m^Tc-anti-CEA IgG-SH in normal mice

**Figure 4:**
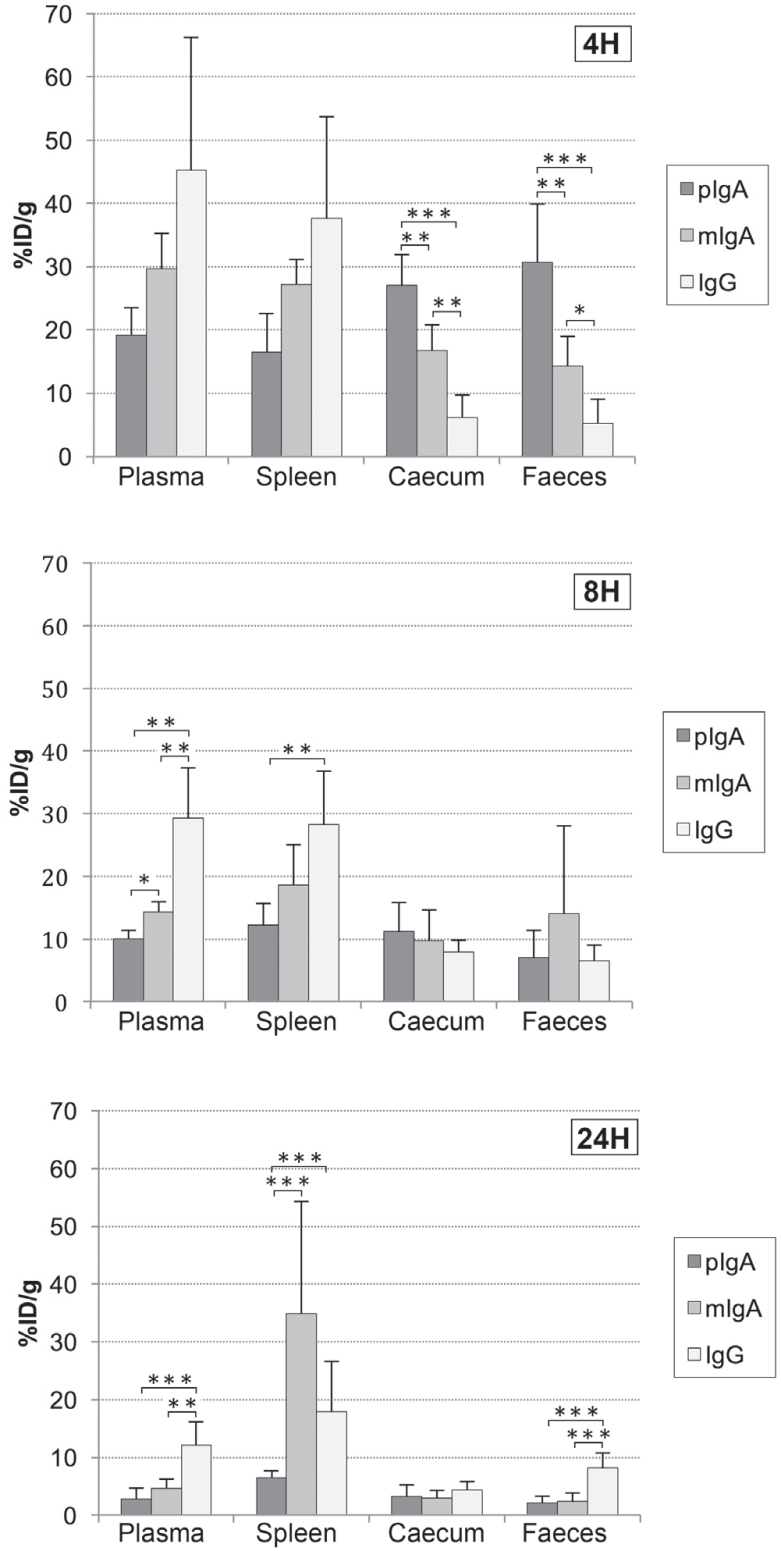
Biodistribution of ^99m^Tc-anti-CEA pIgA-SH, ^99m^Tc-anti-CEA mIgA-SH and ^99m^Tc-anti-CEA IgG-SH Representation of radiolabeled Ig biodistribution at 4, 8 and 24 h post-injection in selected organs: plasma, spleen, caecum and Faeces. Values are expressed as the percentage of injected (intravenous; IV) dose per gram %ID/g and represent the means ± S.D. of the %ID/g. *: *p* value < 0.05 ; **: *p* value < 0.01 ; ***: *p* value < 0.005.

Comparison of the *in vivo* biodistribution kinetics of ^99m^Tc-anti-CEA polymeric IgA-SH, ^99m^Tc-anti-CEA monomeric IgA-SH and ^99m^Tc-anti-CEA IgG-SH in healthy BALB/c mice are presented for all studied organs in Tables 1–3, respectively, and for selected organs in Figure 4.

**Table 1:** Biodistribution of ^99m^Tc-anti-CEA IgA polymeric-SH in healthy Balb-c mice at 4, 8, 18, 24 and 48h, expressed as the percentage of injected (intravenous; IV) dose per gram, %ID/g (values represent means ± S.D. of the %ID/g)

	4h	8h	18h	24h	48h
	(n=4)	(n=4)	(n=4)	(n=8)	(n=4)
**Heart**	5.4±2.5	3.0±0.3	2.4±0.1	1.9±0.8	1.2±0.4
**Brain**	0.33±0.09	0.22±0.04	0.10±0.03	0.11±0.04	0.05±0.01
**Lungs**	7.8±2.7	5.2±0.7	3.4±1.1	3.3±1.1	2.3±1.0
**Kidneys**	21.4±6.9	16.0±5.1	11.6±6.1	11.0±6.6	7.9±4.6
**Spleen**	16.5±6.0	12.2±3.5	6.9±2.7	6.5±1.1	3.9±1.7
**Stomach**	2.7±1.5	1.1±0.4	1.5±1.3	0.7±0.3	0.5±0.2
**Small intestine**	5.2±1.0	3.2±0.6	1.5±0.7	1.1±0.4	0.6±0.2
**Caecum**	27.0±4.9	11.2±4.5	2.8±1.7	3.2±2.1	1.4±0.7
**Colon**	6.7±2.0	3.4±1.3	1.5±0.8	1.1±0.4	0.7±0.4
**Liver**	66.4±19.4	49.0±18.4	19.2±3.9	24.4±11.0	16.9±7.6
**Muscle**	1.6±0.5	1.4±0.6	0.9±0.5	0.6±0.2	0.5±0.3
**Blood cells**	3.3±0.8	2.0±0.5	4.6±2.0	2.1±1.2	1.3±0.5
**Plasma**	19.2±4.3	10.0±1.3	1.3±0.5	2.8±1.9	0.9±0.4
**Faeces**	30.6±9.3	6.9±4.4	3.9±2.6	2.1±1.2	0.9±0.6
**Mesenteric Ganglion**	4.9±3.1	3.0±1.6	1.05±0.3	1.8±1.7	0.5±0.2

**Table 2:** Biodistribution of ^99m^Tc-anti-CEA IgA monomeric-SH in healthy Balb-c mice at 4, 8, 18, 24 and 48h, expressed as the percentage of injected (IV) dose per gram, %ID/g (values represent means ± S.D. of the %ID/g)

	4h	8h	18h	24h	48h
	(n=4)	(n=4)	(n=3)	(n=7)	(n=4)
**Heart**	6.0±0.8	3.4±0.3	3.4±1.4	1.7±0.8	1.6±0.3
**Brain**	0.44±0.06	0.28±0.04	0.17±0.11	0.12±0.04	0.07±0.02
**Lungs**	7.8±1.6	5.3±0.7	5.8±3.0	3.4±1.2	3.0±1.3
**Kidneys**	20.0±4.0	15.8±6.0	16.2±9.2	12.1±6.9	9.8±3.5
**Spleen**	27.2±4.0	18.6±6.5	52.6±14.1	34.8±19.5	41.4±8.7
**Stomach**	2.7±1.7	0.9±0.4	0.9±0.7	1.0±0.7	0.8±0.5
**Small intestine**	1.9±1.8	3.1±0.5	2.2±1.2	1.9±0.6	1.5±0.4
**Caecum**	16.7±4.0	9.8±4.8	3.9±2.2	3.0±1.3	2.1±1.3
**Colon**	4.7±1.2	3.0±1.3	1.8±1.0	1.9±1.1	2.8±2.7
**Liver**	50.6±12.4	34.7±11.5	32.3±8.7	24.1±8.1	21.3±4.4
**Muscle**	1.7±0.6	1.5±0.8	1.7±1.2	0.8±0.6	0.5±0.1
**Blood cells**	4.6±2.4	3.1±1.7	1.5±0.8	1.3±0.7	2.2±0.6
**Plasma**	29.6±5.7	14.3±1.5	6.5±1.8	4.7±1.6	2.0±0.9
**Faeces**	14.3±4.6	14.1±13.9	4.7±2.9	2.4±1.5	2.6±1.9
**Mesenteric Ganglion**	4.5±2.9	3.0±0.7	2.0±0.8	1.4±0.3	0.8±0.2

**Table 3:** Biodistribution of ^99m^Tc-anti-CEA IgG-SH in healthy Balb-c mice at 4, 8, 18, 24 and 48h, expressed as the percentage of injected (IV) dose per gram, %ID/g (values represent means ± S.D. of the %ID/g)

	4h	8h	18h	24h	48h
	(n=4)	(n=4)	(n=4)	(n=4)	(n=4)
**Heart**	9.8±4.8	8.9±1.7	4.0±2.1	3.6±1.6	2.7±1.3
**Brain**	0.61±0.39	0.58±0.07	0.28±0.22	0.23±0.08	0.21±0.10
**Lungs**	16.5±9.3	16.0±6.0	7.9±3.4	6.9±2.0	5.0±1.3
**Kidneys**	28.2±13.9	29.5±3.3	16.3±7.2	15.6±6.6	10.9±5.0
**Spleen**	37.6±16.2	28.3±8.5	18.9±13.2	17.9±8.7	12.6±4.2
**Stomach**	3.3±2.7	3.9±0.3	1.3±0.4	1.6±0.7	1.1±0.3
**Small intestine**	5.3±2.3	4.9±0.7	3.5±1.6	3.2±0.6	1.6±0.4
**Caecum**	6.1±3.6	7.9±1.9	3.7±2.1	4.4±1.4	2.0±0.3
**Colon**	3.8±2.0	4.5±0.2	2.2±1.0	3.2±1.2	1.5±0.5
**Liver**	29.8±16.3	45.5±16.1	18.6±7.3	21.4±4.5	15.7±2.0
**Muscle**	1.6±1.0	3.3±0.9	1.2±0.7	1.2±0.7	0.9±0.5
**Blood cells**	6.6±2.7	6.8±4.5	1.9±0.9	1.6±0.5	1.6±0.7
**Plasma**	45.3±21.0	29.3±8.0	13.7±7.9	12.1±4.1	8.0±1.8
**Faeces**	5.2±3.7	6.5±2.5	2.9±0.9	8.1±2.7	1.8±0.5
**Mesenteric Ganglion**	3.6±3.6	5.9±1.9	2.1±1.4	2.8±2.3	2.0±1.5

Global analysis (i.e. whole-organ study) of radioactivity uptake (percentage of the injected dose per gram of tissue: %ID/g) showed a significant (p<0.002) difference among the three Ig types at 8 h, 24 h and 48 h. However, although no significant difference was observed in global analysis at 4 h among the three Ig types, an important difference in the radioactivity uptake was observed between the caecum and faeces. At this early time, high uptake was measured, in the caecum and faeces, of ^99m^Tc-anti-CEA pIgA-SH (27.0 ± 4.9 and 30.6 ± 9.3 %ID/g, respectively) and, to a lesser extent, ^99m^Tc-anti-CEA mIgA-SH (16.7 ± 4.0 and 14.3 ± 4.6 %ID/g, respectively). In contrast, the uptake of ^99m^Tc-anti-CEA IgG-SH was lower (6.1 ± 3.6 and 5.2 ± 3.8 %ID/g, respectively). Eight hours’ post-injection, significant differences among the three Ig types were observed in the plasma and blood-rich tissues (heart, spleen, lungs). Higher radioactivity was observed with IgG (in plasma: 29.3 ± 8.0 and 12.1 ± 4.1 %ID/g at 8 h and 24 h, respectively), than with mIgA (14.3 ± 1.5 and 4.7 ± 1.6 %ID/g, respectively) or pIgA (10.0 ± 1.3 and 2.8 ± 1.9 %ID/g, respectively). Furthermore, beyond 18 h, strong radioactivity uptake was observed for mIgA in the spleen (at 18 h: 52.6 ± 14.1 %ID/g; at 48 h: 41.4 ± 8.7 %ID/g), whereas no uptake was seen for pIgA or IgG. High radioactivity uptake was also recorded in the liver for all three Ig types. This uptake was more important for pIgA at 4 h (pIgA: 66,4 ± 19,4 %ID/g, mIgA: 50,6 ± 12,4 %ID/g, IgG: 29,8 ± 16,3 %ID/g) but the difference among the three Ig types was not significant.

### Biodistribution of ^99m^Tc-anti-CEA pIgA-SH and ^99m^Tc-irrelevant pIgA-SH in a mouse colorectal tumour model

According to the biodistribution results in normal mice (pIgA-SH higher uptake in the caecum), biodistribution studies of ^99m^Tc-anti-CEA pIgA-SH at 4 h and 8 h were performed in a mouse CRC orthotopic graft model (Table 4) and compared with irrelevant IgA (^99m^Tc-anti-peanut pIgA-SH) and healthy nude mice to evaluate the *in vivo* CEA affinity of IgA. All of the mice included in the studies had macroscopic colorectal tumours in the caecum observed after dissection. A notable accumulation of radioactivity was found in the “faeces-removed” tumoural caecum (15.8 ± 5.5 and 18.5 ± 8.5 %ID/g at 4 h and 8 h, respectively), but this radioactivity uptake was not significantly different from that of the two controls (^99m^Tc-irrelevant pIgA-SH: 16.7 ± 6.9 and 11.5 ± 6.5 %ID/g; nude mouse control: 7.9 ± 2.5 and 19.9 ± 1.4 %ID/g). The pattern of distribution of ^99m^Tc-anti-CEA pIgA-SH between the different organs in the mouse colorectal tumour model was similar to that observed in healthy mouse. However, a significant uptake in the lungs of ^99m^Tc-anti-CEA pIgA-SH was observed after as early as 4 h after injection, and doubled at 8 h (at 4 h: 42.8 ± 59.4 %ID/g; at 8 h: 97.6 ± 29.5 %ID/g). This uptake was significantly more intense (p<0.001) than the low fixation observed in the lungs at 4 h or 8 h after the injection of irrelevant ^99m^Tc-pIgA-SH, as well as in the nude mouse control. For some animals (n = 1–3, depending on group), a tumour was present in the peritoneal cavity opposite the injection site. On these cases, uptake was measured and found to be significantly higher with anti-CEA pIgA than with irrelevant pIgA (2.9 ± 1.4 and 1.5 ± 0.5 %ID/g, respectively; p = 0.0479).

**Table 4:** Biodistribution of ^99m^Tc-pIgA-SH in nude mice bearing intracaecal tumours, and in nude healthy mice at 4h and 8h, expressed as the percentage of injected dose per gram, %ID/g (values represent means ± SD of the %ID/g)

	Nude mice bearing intracecal tumours				Nude healthy mice	
	Anti-CEA pIgA		Irrelevant pIgA		Anti-CEA pIgA	
	4h	8h	4h	8h	4h	8h
	(n=6)	(n=6)	(n=6)	(n=6)	(n=4)	(n=4)
**Heart**	2.5±0.6	2.4±1.2	2.2±0.7	1.3±0.08	3.2±0.6	2.1±0.7
**Brain**	0.24±0.09	0.16±0.04	0.20±0.06	0.11±0.01	0.23±0.08	0.15±0.03
**Lungs**	42.8±59.4	97.6±29.5	3.8±1.5	4.8±1.3	13.5±10.4	19.0±6.6
**Kidneys**	16.0±2.3	17.1±4.7	16.6±5.8	10.4±1.5	16.0±6.0	12.1±0.1
**Spleen**	17.6±12.4	14.5±7.8	9.9±4.9	20.9±8.1	8.2±2.3	7.1±1.3
**Stomach**	1.8±1.0	1.7±0.8	1.3±0.8	0.9±0.3	1.9±0.7	1.9±1.5
**Small intestine**	4.6±1.7	3.6±2.9	4.9±2.1	1.8±0.4	4.7±2.0	3.2±0.2
**Caecum (empty)**	15.8±5.5	18.5±8.5	16.7±6.9	11.5±6.5	7.9±2.5	19.9±1.4
**Colon**	6.4±2.6	3.4±1.8	7.2±4.9	3.2±0.8	10.0±0.6	4.4±0.7
**Liver**	50.5±20.3	46.3±21.8	50.3±7.2	41.9±9.2	34.4±1.2	40.2±3.3
**Muscle**	0.7±0.3	0.9±0.2	0.8±0.2	0.8±0.3	0.9±0.2	0.6±0.1
**Blood cells**	3.1±0.8	2.3±0.9	2.5±1.2	1.3±0.2	2.2±1.3	0.8±0.1
**Plasma**	9.8±2.7	8.2±5.1	7.9±2.2	4.4±0.4	9.7±2.1	5.6±0.6
**Feces**	34.2±24.0	13.3±9.5	34.9±22.9	9.7±6.2	35.2±12.9	35.0±10.1

### Pathological analysis of the caecum and lungs

**Figure 5:**
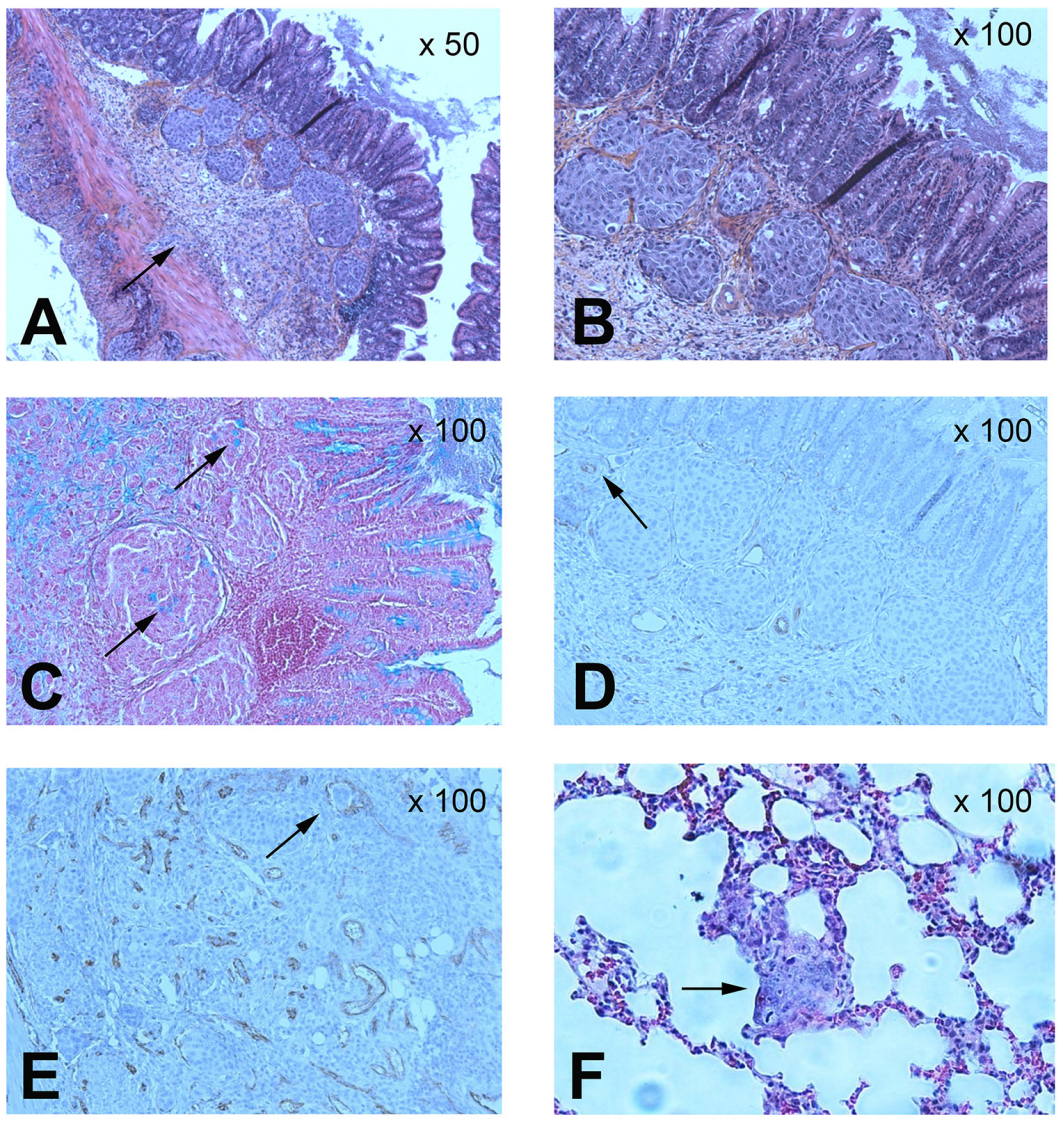
Pathological analysis of caecum and lungs in the tumour mouse model Histochemical analysis of colorectal orthotopic grafts: **(A)** (magnification x50) and **(B)** (x100): haematoxylin, eosin and safran (HES) analysis. **(C)** (x100): mucus cells with Alcian Blue staining. **(D)** (x100): vessels staining with CD31. **(E)** (x100): tumour cells detected in caecum tumour vessels with CD31 staining. Lungs histochemical analysis: **(F)** (x100): HES analysis.

The caecum and lungs in the mouse colorectal tumour model were sliced and analysed using coloration techniques and immunohistochemistry (Figure 5). HES analysis confirmed the presence of tumour mass in the caecal wall, particular in the sub-mucosa (Figure 5A and 5B). Moreover, the tumour cells could secrete mucus, as revealed by Alcian blue staining (black arrows in Figure 5C). The tumours were vascularised, and CD31-positive endothelial cells were detected within the tumour mass (black arrows in Figure 5D). These data confirmed that the model mimics a mucosal human adenocarcinoma. CD31 analysis of the tumour mass revealed that tumour cells were present within tumour vessels (black arrows in Figure 5E). HES analysis in the lungs highlighted the presence of thick and large cells within the pulmonary parenchyma, corresponding to lung metastases (Figure 5F) and explaining the strong uptake of anti-CEA pIgA in this organ (at 4 h: 42.8 ± 59.4 %ID/g; at 8 h: 97.6 ± 29.5 %ID/g).

### MicroSPECT/CT imaging of normal SCID mice and tumour bearing SCID or Tsg SCID-CD89 mice with ^99m^Tc-anti-CEA pIgA-SH, ^99m^Tc-irrelevant pIgA-SH and ^99m^Tc-anti-CEA IgG-SH

Biodistribution of ^99m^Tc-anti-CEA pIgA-SH, ^99m^Tc-irrelevant pIgA-SH and ^99m^Tc-anti-CEA IgG-SH was subsequently evaluated 4 h post-injection by microSPECT/CT in normal SCID mice and in SCID and Tsg SCID-CD89 colorectal tumour model (n=3 for each Ig and mice model).

**Figure 6:**
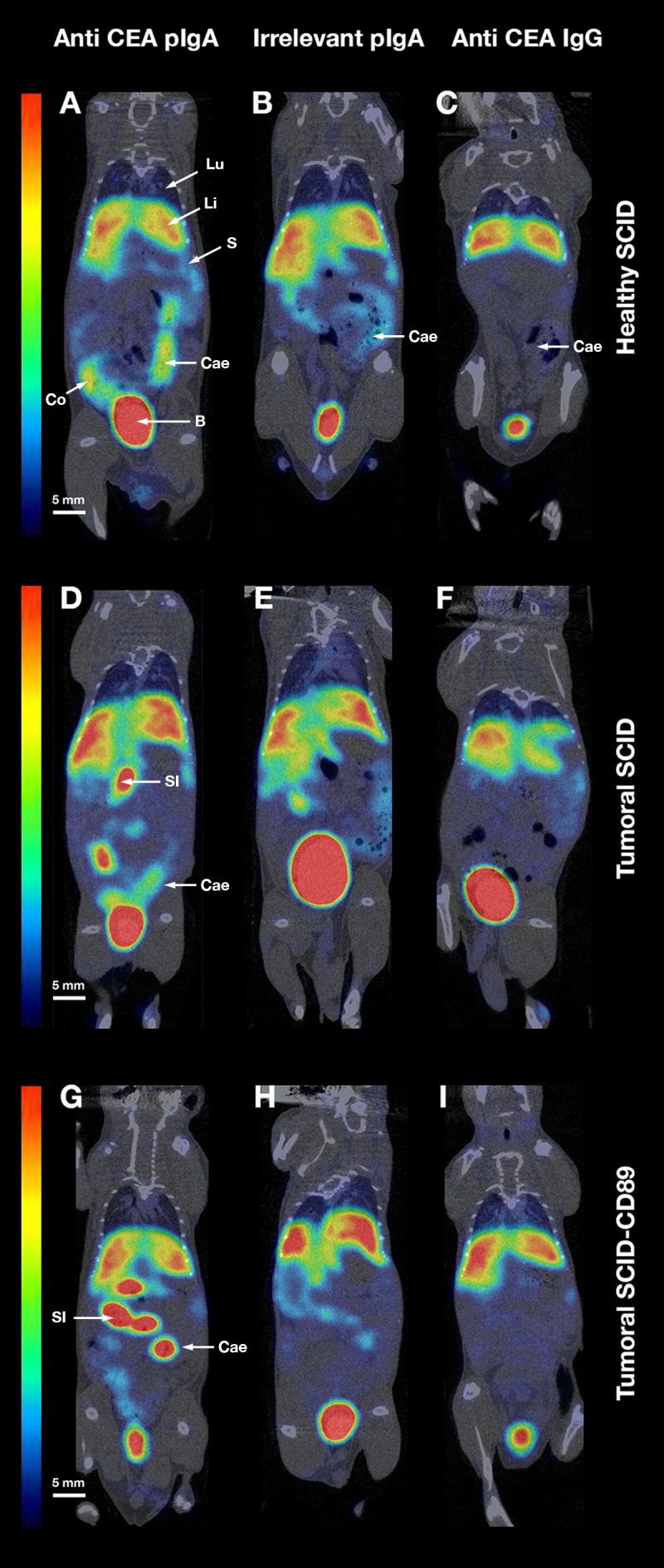
MicroSPECT/CT data MicroSPECT/CT imaging of normal SCID mice **(A-B-C)** and tumour bearing SCID **(D-E-F)** or Tsg SCID-CD89 mice **(G-H-I)** with ^99m^Tc-anti-CEA pIgA-SH (A-D-G), ^99m^Tc-irrelevant pIgA-SH (B-E-H) and ^99m^Tc-anti-CEA IgG-SH (C-F-I). Tumoural mice were grafted by direct orthotopic cell microinjection of human colon carcinoma. Imaging studies were performed 6 weeks after the cell-microinjection procedure. Each mouse received 50-60 MBq of ^99m^Tc-Ig. SPECT/CT images were acquired 4 h post-injection for 30 min. Representative coronal slice (80 μm thickness) of microSPECT/CT acquisition in the different mice models/conditions: - Healthy SCID after injection of ^99m^Tc-anti-CEA pIgA-SH (A), ^99m^Tc-irrelevant pIgA-SH (B), ^99m^Tc-anti-CEA IgG-SH (C) - Tumoural SCID after injection of ^99m^Tc-anti-CEA pIgA-SH (D), ^99m^Tc-irrelevant pIgA-SH (E), ^99m^Tc-anti-CEA IgG-SH (F) - Tumoural Tsg SCID-CD89 after injection of ^99m^Tc-anti-CEA pIgA-SH (G), ^99m^Tc-irrelevant pIgA-SH (H), ^99m^Tc-anti-CEA IgG-SH (I) Principal organs were indicated with a white arrow and letters: B = Bladder, Cae = Caecum, Co = Colon, Li = Liver, Lu = Lung, S = Spleen, SI = Small Intestine.

In healthy mice, for the three Ig, an intense uptake was observed in the liver as highlighted also in the biodistribution studies. Furthermore, an intense uptake was observed in the digestive tract after injection of anti-CEA pIgA and to a lesser extent of irrelevant pIgA, whereas a low uptake of the anti-CEA IgG was revealed (Figure 6A–6C). Total digestive activity was quantified and compared to whole body, for ^99m^Tc-anti-CEA pIgA, ^99m^Tc-irrelevant pIgA and ^99m^Tc-anti-CEA IgG and represented 32 ± 6%, 19 ± 8% and 8 ± 1% respectively.

In engrafted mice, a high uptake was observed in the liver for the three Ig. An intense uptake was highlighted in the digestive tract after ^99m^Tc-anti-CEA pIgA injection (Figure 6D and 6G), compared to the low uptake of ^99m^Tc-anti-CEA IgG (Figure 6F and 6I). Nevertheless, the whole digestive tract uptake of ^99m^Tc-anti-CEA pIgA didn’t significantly differ between healthy and engrafted mice (32 ± 6% and 30 ± 9% respectively).

In both healthy and engrafted mice, an intense uptake of ^99m^Tc-anti-CEA IgA was also noticed in the gallbladder (n = 9, data not shown) whereas no significant uptake was ever observed in mice injected with the ^99m^Tc-anti-CEA IgG (n = 9, data not shown). This result is consistent with the physiological hepato-biliary pathway of IgA.

When the uptake of IgA observed in engrafted SCID mice (Figure 6D and 6E) was compared to the uptake observed in engrafted Tsg SCID-CD89 mice (Figure 6G and 6H), the contribution of FCαRI does not appear to be determinant. No significant increase uptake was observed in engrafted Tsg SCID-CD89 mice.

**Figure 7:**
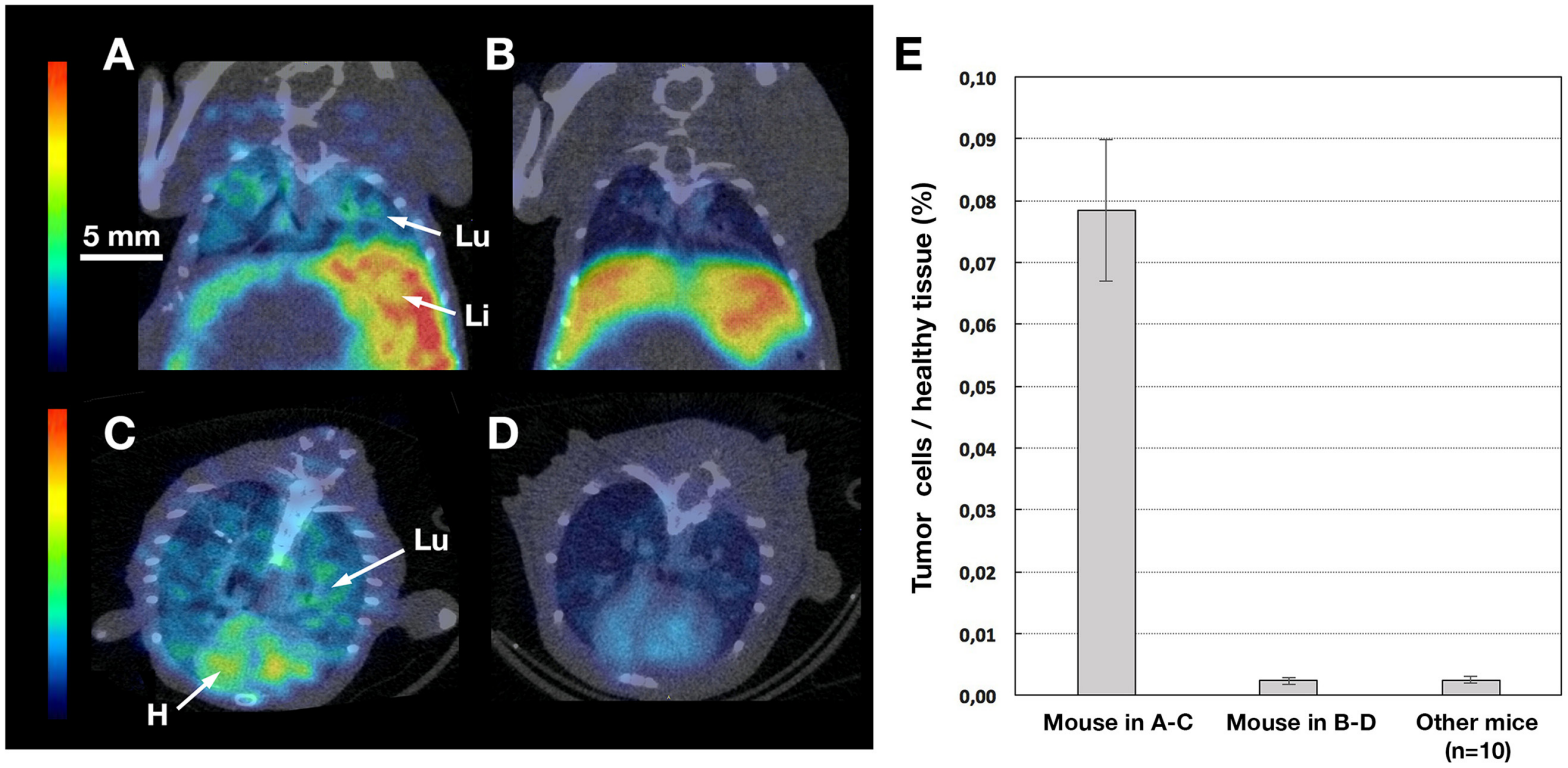
MicroSPECT/CT and qPCR pulmonary data MicroSPECT/CT imaging of tumour bearing SCID with ^99m^Tc-anti-CEA pIgA-SH **(A-B-C-D)** and qPCR of pulmonary sample **(E)**. Tumoural mice were grafted by direct orthotopic cell microinjection of human colon carcinoma. Imaging studies were performed 6 weeks after the cell-microinjection procedure. Each mouse received 50-60 MBq of ^99m^Tc-anti-CEA pIgA-SH. SPECT/CT images were acquired 4 h post-injection for 30 min. 80 μm thickness slice in coronal (A-B) and axial plans (C-D) of a representative view of tumour bearing SCID mice. Principal organs were indicated with a white arrow and letters: H = Heart, Li = Liver, Lu = Lung. The mouse in A-C was in a higher metastasis evolution (peritoneal carcinosis) than the mouse in B-D, confirmed by qPCR analysis on pulmonary samples (E). The ^99m^Tc-anti-CEA pIgA-SH uptake is diffuse and significantly higher in the lung of the mouse in A-C than in B-D mouse.

As the engrafted mouse models (NUDE, SCID, Tsg SCID-CD89) developed metastases in both liver and lungs, colonisation was evaluated by a sensitive real-time (TaqMan) PCR technique and compared with microSPECT/CT data. The percentage of metastases infiltration in the lungs, for both engrafted SCID and Tsg SCID-CD89 mice, reached the level of 0.0003% to 0.0052% of the lung tissue (Figure 7E). At this early stage of metastases spreading, the ^99m^Tc-anti-CEA IgA failed to detect significant uptake by microSPECT/CT imaging (mean value in pulmonary Region of Interest (ROI): 0,5 ± 0,1 in Figures 6D-6G and 7B-7D). However, when the tumour infiltrate represented higher colonisation (0.08% ± 0.01%) of the pulmonary tissue, as observed in one mouse, a significant diffuse uptake of ^99m^Tc-anti-CEA IgA was detected in the lung (mean value in pulmonary ROI: 1,4 in Figure 7A-7C) but no pulmonary nodule was identified in microSPECT/CT slice, while the imaging resolution was about 1 mm. In the liver, despite a high level of colonisation quantified by PCR (data not shown) and also observed macroscopically, the uptake was important but not significantly different from healthy mice.

## DISCUSSION

This work reported anti-CEA IgA radiolabelling with ^99m^Tc to evaluate IgA biodistribution compared with IgG, and to estimate IgA tumour-targeting potency.

This study focused on IgA, a class of Ig that to date has been under-studied compared with IgG. Presently, IgG is the most widely used antibody in therapy and clinical trials. However, some functional limitations of IgG, such as inadequate tissue accessibility and pharmacokinetics, and impaired interactions with effector cells, have been highlighted [21]. IgA could represent a promising alternative to IgG, particularly to target mucosal tumours, considering that IgA constitutes the major Ig class at the mucosal surface.

To develop a soft ^99m^Tc radiolabelling method applicable to IgA, which preserves the biological functions of antibody and is suitable for low-concentration samples of mAbs, we selected the method initially developed by Biechlin *et al.* [22] and subsequently optimised and evaluated by Carpenet *et al.* [20]. This method involved two steps: Ig derivatisation with a synthon, 2-IT, and Ig-SH radiolabelling by an indirect method using the ^99m^Tc-tricarbonyl core. The ^99m^Tc-tricarbonyl core is a complex with high kinetic stability and an excellent precursor for radiolabelling various biomolecules, especially Igs. To functionalise Ig by adding SH- groups under milder conditions, and to obtain high radiolabelling yields, the 2-IT synthon was used. Our results confirmed the significant additional value of the derivatisation step for IgA, as previously observed for IgG [19], allowing an RCP > 95%. High RCP enables radiolabelled Ig administration without an additional purification step. The stability of radiolabelled IgA was checked over 24 h in PBS and murine plasma. Studies in PBS showed that IgA radiolabelling was stable. In murine plasma, the RCP decreased progressively over 24 h, suggesting slight degradation comparable with that seen in IgG stability studies. Our stability results were also consistent with published data from Ig studies using tricarbonyl core (loss of 10–15%) [23, 24] or direct labelling after disulphide bond reduction [25].

Western blot analyses indicated that the radiolabelling process did not alter the IgA global molecular structure. Affinity studies confirmed that radiolabelled IgA showed a well-preserved binding affinity (Kd ≈ 25 nM). Concordant Kd were obtained with the two affinity determination methods. All of these *in vitro* studies suggest that radiolabelling preserves IgA functionality.

Biodistribution studies in healthy mice have compared radiolabelled pIgA, mIgA and IgG, all carrying the same variable domains directed against the CEA antigen. The mIgA presented an intermediate fixation profile between the profiles shown by pIgA and IgG, except in the spleen. Although the liver is the main organ involved in IgA removal, participation of the spleen in clearance was already observed also in some cirrhosis patients and was almost invariably concurrent with normal parameters [26]. Compared with IgG, a greater and more rapid fixation (at 4 h) was observed in the caecum and faeces for pIgA and, to a lesser extent, mIgA. This study confirms that pIgA has a strong and fast tropism for the caecum at the *lamina propria,* as previously described in the literature. Rapid secretion in the gut lumen and high concentration in the faeces (4 h) was observed; however, for IgG, radioactivity was maximal in the faeces at 24 h. Significant differences between pIgA/mIgA and IgG were also observed in the plasma and blood-rich tissues. The plasma retention of mIgA and pIgA decreased rapidly, which is consistent with their well-known short sera half-life [27]. This appears to be balanced by their rapid rise in concentration, as observed in faeces, in accordance with the redistribution in the mucosal membrane. In mouse models, the short serum half-life of IgA antibodies seems to be partially caused by rapid clearance by the asialoglycoprotein receptor (ASGPR) recognising terminal galactose residues of IgA [28, 29], even if this clearance pathway is more addressed to IgA2 than IgA1. In contrast, IgG is characterised by a relatively long biological half-life in the bloodstream. Unlike IgA, IgG binds to the neonatal receptor FcRn, expressed on vascular endothelial cells, macrophages and monocytes. This interaction could be one explanation for the longer serum half-life of IgG [30]. Recently, by molecular fusion of an albumin-binding domain (ABD), the half-life and *in vivo* serum exposure of ABD-modified IgA was found to increase significantly due to indirect targeting of the FcRn pathway [31]. Concerning the liver, a high uptake of the three Ig isotypes studied herein was observed, explained in part by the expression of the ASGPR on the hepatocyte surface, but also by engagement of IgA in the hepatobiliary system involved in IgA secretion in the bile duct [32]. pIgA is transported from blood to bile without degradation [33], through hepatocytes by the pIgR. SIgA is released in the bile. Most of the pIgA that are transported from blood to bile in the liver are synthesized by plasma cells found in the *lamina propria* of the intestinal mucosa and enters the bloodstream at the thoracic duct. Circulating pIgA is then bound at the sinusoidal front of hepatocytes and transported across the cell, processes mediated by the pIgR. According to Hoppe’s work [34], 36% of ^125^I-pIgA (mouse myeloma producted) administrated by IV in rat, are transported as intact protein in bile over 3h post-injection; the pIgA reaches its highest concentration only 30 to 60 min after injection, and 80% of the total of ^125^I-pIgA are finally secreted in bile by 90 min. MicroSPECT/CT imaging confirmed and highlighted hepatobiliary system as a high uptake was observed in the gallbladder.

To evaluate IgA targeting potency, a CRC tumour model was set up in which human cancer cells were grafted in the mucosal environment. Pathological microscopic analysis clearly revealed a structural glandular architecture of the grafted tumour and the presence of large vacuoles in the WiDr cell line, consistent with muco-secretions in the *lamina propria* layer. Cancer cells invaded the normal caecum, under the muscle layer through the *lamina propria*, to produce protruding polyps in the lumen. Depending on the delay after direct orthotopic cell microinjection, different stages of CRC have been observed from localised tumours to metastases in the lungs. Immunohistochemical analysis in tumours revealed that tumour cells were present within tumour vessels, suggesting cellular dissemination by the vascular system. All of these factors led us to consider colorectal orthotopic grafts as being useful models of human CRC, because they share the same characteristics with human tumours. In humans, in advanced colorectal cancer, metastases are spread in the liver and lung in particular. The engrafted mouse models (NUDE, SCID, Tsg SCID-CD89) mimic metastases colonisation in both liver and lung.

Surprisingly, in *post mortem* biodistribution studies, no significant difference in caecum uptake was observed after the injection of radiolabelled anti-CEA IgA or irrelevant IgA, or in healthy nude mice. Tumours implanted in the external layers of the caecum progress by infiltrating the whole caecum, without an evident limit between the tumoural tissue and the healthy tissue. Caecal tumours were partly necrotic and weakly vascularised in some regions. Furthermore, pIgA, regardless of their antigenicity, have a strong tropism for caecal mucosa. So, the tumour-to-background ratio was not optimal, and radioactive determination of the whole caecum probably hampered tumour-specific signal detection. It is well-known that, in the late stage of tumour differentiation, the majority of CEA molecules may not be reached by IV-injected mAbs because CEA is mostly expressed on the apical side of carcinoma cells hidden by the pseudo-lumen structures of the malignant glands [35]. However, human colon carcinoma cells maintained pIgR expression on the basolateral side on the external part of the pseudo-lumen structures. By transepithelial translocation, only dIgA, and not IgG, can reach the pseudo-lumen to target CEA at this late stage of adenocarcinoma differentiation. Conversely, lung metastases are characterised by an immature stage of differentiation, without any glandular organisation and certainly without CEA-specific polarisation. Thus, it is interesting and important that anti-CEA pIgA uptake was observed in the lungs, at a significantly higher level than that seen after injection of radiolabelled irrelevant IgA, or in healthy nude mice. This result is consistent with the presence of lung metastases, as demonstrated by pathological analysis. Even if no significant uptake was seen in the tumoural caecum with anti-CEA pIgA, this antibody allowed the targeting of lung metastases derived from the initial colorectal tumour. Due to intrinsic mucosal tropism and biomarker affinity, pIgA could very effectively reach its target in the lungs.

The *in vivo* imaging by microSPECT/CT confirm that the anti-CEA pIgA is a suitable tool for the detection of mucosal tumours, particularly in lungs, but *in vivo* imaging is less sensitive (1 mm resolution) and uptake was detectable only for a intermediate tissue colonisation (0.08% ± 0.01%). In the liver, partly because of the high natural uptake of IgA (by the hepato-biliary pathway), the anti-CEA pIgA seems not be well adapted in detecting metastases, hide by the strong uptake background, despite a massive metastases colonisation quantified by qPCR (up to 0,9%, data not show).

The FcαRI is a critical receptor to mediate cytotoxicity effect, but its involvement in pIgA biodistribution is still unclear. To investigate this pathway, microSPECT/CT studies were conducted in transgenic mice Tsg SCID-CD89. No significant increase uptake was observed in Tsg SCID-CD89 mice compared to SCID mice, neither in digestive tract, neither in lungs. This result suggests that the distribution of the IgA molecule could occur without active transport linked to the FcαRI receptor by infiltration of myeloid cells at the mucosal site, particularly in digestive tract. In pulmonary mucosal, further investigations are necessary to clarify this preliminary report.

In conclusion, monomeric and polymeric IgA were efficiently and indirectly radiolabelled with ^99m^Tc, using limited amounts of antibody. *In vitro* studies showed that IgA radiolabelling was stable and did not alter IgA functionality. Biodistribution studies in normal BALB/c mice, of ^99m^Tc-anti-CEA mIgA-SH and ^99m^Tc-anti-CEA pIgA-SH, confirmed their shorter serum half-life and rapid and strong mucosal tropism of pIgA and, to a lesser extent, mIgA. High lung uptake of ^99m^Tc-anti-CEA pIgA-SH in the mouse tumour model suggested efficient targeting potency of pIgA, even though a significant and specific uptake in caecum was not observed. As SIgA is resistant to intestinal degradation, it would be interesting to explore SIgA biodistribution after oral administration. SIgA reverse transcytosis, mediated by epithelial M cells, remains understudied, but in humans several receptors seem to be involved (i.e. Dectin-1, DC-SIGN [36], CD71 [37]). Finally, this work represents a first step toward IgA development to envisage, in the near future, diagnostic imaging tools and therapeutic IgA-based strategies targeting tumours in mucosal epitheliums.

## MATERIALS AND METHODS

All of the chemicals and reagents were obtained from Sigma-Aldrich (Saint-Quentin Fallavier, France) unless otherwise specified.

### Ig production

All of the Ig types were produced by B Cell Design Society (Limoges, France). Monomeric and polymeric anti-CEA (relevant) or anti-peanut (irrelevant) human chimeric IgA1 were synthesised using HAMIGA™ technology [18]. For *in vitro* studies, a mpIgA was used; however, for *in vivo* studies, the enriched fraction of the mIgA or pIgA form was tested separately (purities of 95% and 85%, respectively). Anti-CEA human recombinant IgG1 was synthesised after cloning the variable regions of the heavy and light chains of anti-CEA human chimeric IgA1. It was then produced in human embryonic kidney cells (HEK 293-6E; NRC, Quebec, Canada). All of the antibodies were purified by affinity chromatography using a Tricorn Column 5/100 with protein A-Sepharose at a flow rate of 1.0 mL/min (GE Healthcare, Waukesha, WI, USA) and were eluted with glycine (0.1 M pH 2.7) equilibrated in Tris/base (1.0 M). Subsequently, IgA and IgG were dialysed against PBS by centrifugation (1,000 × *g*, 15 min) using Amicon 30 kDa (Millipore, Saint-Quentin, France).

For imaging experiment, anti-CEA IgG was purchased (1105 clone, Fisher Scientific, Elancourt, France).

The protein concentrations were determined before and after radiolabelling using bicinchoninic Micro BC Assay^®^ kits (Fisher Scientific, Elancourt, France), using bovine serum albumin (BSA) as a standard with quantification limits of 2.5 and 100 μg/mL.

### Cell culture

Two human colorectal cell lines were used: WiDr, a primary adenocarcinoma of the rectosigmoid [38], and DLD1, a colorectal glandular carcinoma [39]. WiDr, which are CEA-expressing cells, and DLD1, which are CEA-negative cells [40], were purchased from ATCC (Manassas, VA, USA). The two cell lines were routinely grown in RPMI and minimum essential medium, respectively, supplemented with 10% foetal calf serum, 1% sodium pyruvate, and 1% penicillin (100 U/mL)-streptomycin (100 μg/mL). For WiDr, the medium was also supplemented with 1% glutamine and 1% nonessential amino acids.

### Ig radiolabelling with [^99m^Tc(CO)_3_(H_2_O)_3_]^+^

The radiolabelling method used has been described previously [20]. Briefly, the first step was thiol-derivatisation of Ig with 2-IT. Next, 0.5 to 2.2 nmol IgA and IgG (300 μL in PBS) were incubated with 2-IT (3.8 μM, 25°C, 120 min). The solutions were purified by size exclusion chromatography. The number of thiol groups was determined by a micromethod using Ellman’s reagent (5.5’-dithiobis-2-nitrobenzoic acid, DTNB). The second step was synthesis of the tricarbonyl precursor [^99m^Tc(CO)_3_(H_2_O)_3_]^+^. Next, 0.8–1 mL of freshly eluted [Na^99m^TcO_4_] (CisBio, Saclay, France) in fixed activities (2,220–3,700 MBq) was added to the IsoLink^®^ kit (Covidien, Petten, The Netherlands) and incubated for 25 min at 100°C. RCP analysis was performed by thin-layer chromatography (TLC) using two systems to separate the [^99m^Tc(CO)_3_(H_2_O)_3_]^+^ from free [^99m^Tc]-pertechnetate, reduced ^99m^Tc and hydrolysed [^99m^Tc(OH)_n_(H_2_O)_y_]: (1) Baker Flex Aluminium oxide strips (JT Baker Inc., Phillipsburg, NJ, USA) - methanol/hydrochloric acid (95/5 v/v); (2) Instant thin layer chromatography–silica gel (ITLC-SG, Varian, Les Ulis, France) - methanol. The ^99m^Tc-Isolink^®^ labelling yields were superior to 98%. The third and last step was the radiolabelling of native or derivatised Ig with [^99m^Tc(CO)_3_(H_2_O)_3_]^+^. A total of 0.5–2.2 nmol of non-derivatised IgA or thiol derivatised IgA (IgA-SH), or 1.5 nmol IgG-SH in 300 μL of PBS, was incubated for 120 min (25°C) with 150 μL (148–185 MBq) of a ^99m^Tc-tricarbonyl solution, previously neutralised to pH 7.0 (0.5 M HCl). RCP was determined by TLC with ITLC-SG/NaCl 0.9%.

### *In vitro* stability of ^99m^Tc-IgA-SH

The stability of radiolabelled IgA-SH was checked for 24 h, in PBS (4°C and 25°C) and murine plasma (1/10, 37°C). Aliquots of 5 μL were analysed by TLC (ITLC-SG/NaCl 0.9%) at various time points (1, 2, 4, 16 and 24 h).

### Structural integrity of ^99m^Tc-IgA-SH

SDS-PAGE was performed under non-reducing conditions with the native or labelled Ig. The proteins (1.5–10 μg per lane) and molecular weight standards (Bio-Rad, Hercules, CA, USA) were loaded and resolved with a Mini-Protean TGX Precast Gel using a Bio-Rad apparatus. Proteins were immediately transferred to a PVDF membrane. The membrane was then washed, blocked with a 3% milk phosphate buffer and incubated (90 min, 25°C) in diluted primary antibody solution (1/1,000, goat antihuman IgA-horseradish peroxidase; Southern Biotech, Birmingham, AL, USA). The membrane signals were revealed using the diaminobenzidine (DAB) substrate.

### Affinity of ^99m^Tc-IgA-SH

The binding affinity was evaluated by two complementary methods: in plate with coated CEA antigen and directly on cells expressing CEA antigen.

### Binding affinity by RIA

To evaluate anti-CEA IgA binding to human CEA, removable well plates were coated with 1 μg/well of CEA antigen (AbCys SA, Courtaboeuf, France) and incubated (overnight, 4°C) in PBS at pH 7.4. Next, they were blocked with PBS-gelatine 2% (1 h, 37°C). Relevant (anti-CEA) or irrelevant (anti-peanut) radiolabelled IgA (100 μL, 0.5–16 μg/mL) was added in series to antigen-coated wells. The first series was diluted in PBS-gelatine 0.2% and the second was diluted in PBS-gelatine 0.2% containing unlabelled antibody at 500 μg/mL (non-specific binding). Plates were incubated (2 h, 37°C) and washed three times with PBS at pH 7.4. The radioactivity contained in the wells was determined by gamma counting (Cobra 5003; Canberra Packard, Frankfurt, Germany).

### Binding affinity on cells

Saturation binding studies were assessed according to the method of Lindmo *et al.* [41, 42]. The ^99m^Tc-IgA-SH binding affinity experiments were performed on WiDr (anti-CEA and anti-peanut IgA) and DLD1 (anti-CEA IgA). Samples of cells (1 million cells/250 μL) were first pre-incubated with an anti-mouse CD16/CD32 IgG2Bk (1/250, 25°C, 30 min; BD Biosciences Pharmingen, Maharashtra, India) to block the Fc receptor. They were then pre-incubated with PBS–BSA 3% or with unlabelled antibody (final concentration of 100 μg/mL in PBS–BSA 3%) to saturate the binding sites for nonspecific binding determination (25°C, 90 min). Next, duplicate samples were incubated with ^99m^Tc-IgA-SH increasing concentrations (125 μL, 0.1 to 10–13 μg/mL). After incubation (2 h, 25°C) with continuous rotation, the cells were filtered using Manifold® (Millipore) and washed with PBS. Filter radioactivity was evaluated with a gamma-counter.

### Affinity data analysis

For wells and cell-binding affinity studies, specific binding was evaluated by subtracting nonspecific binding (determined after incubation with unlabelled antibody) from total binding. Data analysis was performed using a Scatchard plot of the binding of ^99m^Tc-anti-CEA IgA-SH, and the Kd, number of antibody binding sites/well or cell, and B_max_ of ^99m^Tc-anti-CEA IgA-SH were determined.

### Biodistribution of ^99m^Tc-anti-CEA IgA-SH and ^99m^Tc-anti-CEA IgG-SH in normal mice

All *in vivo* experiments were performed in accordance with animal ethical rules, and all efforts were made to minimise suffering. The protocol was approved by the Comité Régional d'Ethique sur l'Expérimentation Animale du Limousin (CREEAL). Biodistribution experiments were carried out in 7-week-old male BALB/c mice (Charles River Laboratories, Chatillon-sur-Chalaronne, L'Arbresle Cedex). ^99m^Tc-IgA-SH monomeric, ^99m^Tc-IgA-SH polymeric or ^99m^Tc-IgG-SH (40 MBq, 170 μg for each antibody) was injected intravenously (IV; tail vein). Animals were euthanised, by anaesthesia and cervical dislocation, at different time points after administration (4 h, 8 h, 18 h, 24 h, 48 h post-injection). Selected tissues were excised, rinsed, and weighed, and their radioactivity levels were measured with a gamma-counter. The uptake of radioactivity in these organs was expressed as %ID/g after correcting for radioactive decay at each time point. Blood cells, plasma, and faeces were also collected and measured. Faeces refers to faecal matter collected in the small intestine and colon during dissection. Liver and gallbladder were not separated before counting.

### Biodistribution and tumour uptake of ^99m^Tc-anti-CEA pIgA-SH in nude mice bearing human colon carcinoma

Direct orthotopic cell microinjection was achieved according to the method of Cespedes *et al.* [43].

Seven-week-old female nude mice (athymic nu/nu; Envigo, Gannat, France) were anesthetised with ketamine (80 mg/kg) (Imalgène, Merial, Lyon, France) and xylazine (9.6 μg/kg) (Rompun 2%; Bayer, Lyon, France) to exteriorise their caecum by laparotomy. Next, 2.10^5^ WiDr cells, suspended in 10 μL of PBS in a sterile micropipette (30 G needle, 30 μL syringe), were slowly injected between the mucosa and *muscularis externa* layers of the caecal wall, under a binocular lens with an approximate 30° angle. After injection, the caecum was extensively washed with sterile PBS before reintroduction into the abdominal cavity. The laparotomy was closed by surgical suture. Animals were weighed each day. If animals demonstrated clinical alteration or weight loss, they were euthanised by anaesthesia and cervical dislocation.

### Biodistribution and tumour uptake

Six weeks after the cell microinjection procedure, the animals were divided into two groups. The first group (*n* = 6) received 170 μg of ^99m^Tc-anti-CEA pIgA-SH (IV) and the second (*n* = 6) received 170 μg of irrelevant ^99m^Tc-pIgA-SH (corresponding to 35–37 MBq). Nude mouse controls received the same ^99m^Tc-anti-CEA pIgA-SH. The animals were euthanised by anaesthesia and cervical dislocation at 4 h and 8 h after administration. Finally, the procedure described in biodistribution studies of normal mice was applied. Furthermore, the caecum was longitudinally opened, washed with PBS and counted separately from caecal faeces to evaluate luminal IgA presence. The caeca and lungs were fixed with buffered formalin during radioactive decay (48 h).

### Histological analysis of human colorectal orthotopic grafts

Mouse organs were transferred to 4% formol and included in paraffin after automated cycling of a dehydration system (ASP200S; Leica, Heidelberg, Germany). Next, 4-μm sections were prepared using a microtome. For histological analysis, slides were stained with haematoxylin, eosin and safran (HES analysis) or with Alcian blue (secretion mucus analysis). For vascularisation analysis, CD31 staining was performed using the VENTANA robot from the pathology department of Limoges University Hospital. In each case, a pathologist was solicited to interpret the staining.

### MicroSPECT/CT imaging of normal SCID mice and tumour bearing SCID or Tsg SCID-CD89 mice with ^99m^Tc-anti-CEA pIgA-SH, ^99m^Tc-irrelevant pIgA-SH and ^99m^Tc-anti-CEA IgG-SH

SCID (Envigo, Gannat, France) or Tsg SCID-CD89 mice (J. Leusen, University Medical Center Utrecht, The Netherlands) were grafted as described previously for nude mice. ^99m^Tc-anti-CEA pIgA-SH, ^99m^Tc-irrelevant pIgA-SH or ^99m^Tc-anti-CEA IgG-SH (60 MBq) was injected IV. Anaesthesia induction was achieved using 3% isoflurane and animals were maintained under general anaesthesia with 1,8% isoflurane for the duration of imaging. Air/Oxygen (50%/50%, 1,4 l/minutes) was maintained throughout anaesthesia induction and maintenance. Acquisitions were performed 4 h post-injection with a MicroSPECT/CT (U-SPECT4/CT, MILabs, Utrecht, The Netherlands). Images were acquired during 30 minutes and energy windows were set over the 140 keV peaks (±20%). The SPECT resolution with ^99m^Tc is less than 1 mm. Images were analysed and uptake were quantified in Region of Interest (ROI) with PMOD Software (PMOD Technologies, Zürich, Switzerland).

### qPCR on lung samples

The presence of human tumour cells and metastases within engrafted mouse organs was quantified, by a TaqMan-chemistry based real-time PCR using a primer/probe-combination described previously [44]. Briefly, two days after microSPECT/CT imaging, animals were euthanized, by anaesthesia and cervical dislocation, and lung tissue was excised, two samples (in right lobe, left lobe) of 0.02 g were collected. Sample DNA was extracted using a nucleoSpin Tissue kit (Macherey-Nagel, Hoerdt, France) and quantify on nanodrop quantifier (Ozyme, Montigny, France). A short fragment (467bp) of the α-satellite region of the human chromosome 17 was amplified using a probe (Cr17-probe) 5’-labelled with the reporter fluorescent dye FAM (6-carboxy-fluoresceine) and linked to a non-fluorescent quencher dye TAMRA. The reactions were performed in a Applied Biosystems Step One Plus Real Time PCR, and analysed by the Step One software (v2.3). The PCR were run in 96-well microtiter plates with a final volume of 20 μL, containing 12.5 ng of genomic DNA and 10 nM of each primer. 45 cycles were performed. Each sample was tested twice in parallel. Serial dilutions of human carcinoma cells (WiDR cell line) in mouse lung tissue (0.02 g biopsy) served to define the TaqMan-calibration curves. In parallel, a human-mouse control PCR was set up in order to normalize DNA quantity per sample. A primer/probe combination was designed in the β-actin encoding region, to be absolutely identical both in human and in mouse sequence (β-actin Forward primer: (5’_tctgcgcaagttaggttttg_3’; β-actin reverse primer: (5’_gatcattgctcctcctgagc_3’; FAMβ-actin_probeTAMRA: (5’_tcatactcctgcttgctgatc_3’) and amplified a short (211bp) fragment.

### Statistical analysis

Statistical analyses were performed by applying the nonparametric Kruskal-Wallis test for antibody comparison. The tests were conducted using StatView software (ver. 5.0 ; SAS Institute, Cary, NC, USA). A *p* value < 0.05 was considered to indicate statistical significance.
